# Evaluation of the models handling heterotachy in phylogenetic inference

**DOI:** 10.1186/1471-2148-7-206

**Published:** 2007-11-01

**Authors:** Yan Zhou, Nicolas Rodrigue, Nicolas Lartillot, Hervé Philippe

**Affiliations:** 1 Canadian Institute for Advanced Research. Département de Biochimie, Université de Montréal, Succursale Centre-Ville, Montréal, Québec H3C3J7, Canada; 2 Laboratoire d'Informatique, de Robotique et de Microélectronique de Montpellier. CNRS – Université de Montpellier 2. 161, rue Ada, 34392 Montpellier Cedex 5, France

## Abstract

**Background:**

The evolutionary rate at a given homologous position varies across time. When sufficiently pronounced, this phenomenon – called heterotachy – may produce artefactual phylogenetic reconstructions under the commonly used models of sequence evolution. These observations have motivated the development of models that explicitly recognize heterotachy, with research directions proposed along two main axes: 1) the *covarion *approach, where sites switch from variable to invariable states; and 2) the *mixture of branch lengths *(MBL) approach, where alignment patterns are assumed to arise from one of several sets of branch lengths, under a given phylogeny.

**Results:**

Here, we report the first statistical comparisons contrasting the performance of covarion and MBL modeling strategies. Using simulations under heterotachous conditions, we explore the properties of three model comparison methods: the Akaike information criterion, the Bayesian information criterion, and cross validation. Although more time consuming, cross validation appears more reliable than AIC and BIC as it directly measures the predictive power of a model on 'future' data. We also analyze three large datasets (nuclear proteins of animals, mitochondrial proteins of mammals, and plastid proteins of plants), and find the optimal number of components of the MBL model to be two for all datasets, indicating that this model is preferred over the standard homogeneous model. However, the covarion model is always favored over the optimal MBL model.

**Conclusion:**

We demonstrated, using three large datasets, that the covarion model is more efficient at handling heterotachy than the MBL model. This is probably due to the fact that the MBL model requires a serious increase in the number of parameters, as compared to two supplementary parameters of the covarion approach. Further improvements of the both the mixture and the covarion approaches might be obtained by modeling heterogeneous behavior both along time and across sites.

## Background

Probabilistic methods for phylogenetic inference are based on mathematical models of sequence evolution [1]. In the last 20 years, several approaches have been proposed for developing more sophisticated models, accounting for various properties of substitution processes [2-8]. One of the most well-characterized example of such an improvement is provided by the Rate Across Sites (RAS) model [2], which relaxes the assumption that all sites of a protein or a nucleotide sequence evolve at the same rate. More specifically, the RAS model includes site-specific substitution rates, modeled as random variables following a gamma distribution. It generally has a better fit to the data, and it allows to circumvent certain artefacts in phylogenetic inference [9]. It has been implemented in most maximum-likelihood and Bayesian phylogenetic software, and is now widely used for routine phylogenetic inference. More sophisticated distributions of substitution rates, such as mixtures of gamma distributions [10], further increase the fit of the model to alignments, suggesting that improvements of the RAS model are still possible.

Functional and structural restrictions operating at a given residue may be subject to change over time [11,12], which should be reflected by substitution rates varying not only across sites, but also across time. In this line of thought, Fitch and Markowitz [13] proposed the covarion hypothesis: due to functional restrictions, some codons (the *co*ncomitantly *vari*able cod*ons *or covarions) can accept substitutions at a given time, while others (invariant sites) cannot. Importantly a site can shift from being variable to being invariable (and vice versa) over time. More generally, Philippe and Lopez [14] proposed, instead of covarion-like expression, the term heterotachy (from Greek, meaning "different speed") to describe the fact that sites evolve at different rates across time.

Heterotachy was shown to be frequent in both nucleotide and amino acid sequences [6,15-22]. For instance, up to 95% of the variable sites of cytochrome b have a heterotachous behavior within vertebrates [23]. Importantly, both simulation [24,25] and empirical [26,22,16,28] studies demonstrate that heterotachy may impede phylogenetic inference. This is expected because probabilistic methods are inconsistent when the underlying assumptions of their models are seriously violated. Models that handle heterotachy are thus of prime interest, particularly as larger and larger datasets are used [29].

The initial covarion hypothesis, as formulated by [13], makes an explicit link between site interdependencies and rate shifts, and for that reason, is not easy to implement. As a more tractable alternative, Tuffley and Steel [30] proposed a site-independent mathematical version of the covarion idea, which was later implemented in a Bayesian framework [6]. In Tuffley and Steel's covarion model, the substitution history at each site unfolds according to a doubly stochastic process: a classical first-order Markov process of substitution among the 4 nucleotide bases, or the 20 amino-acids, whose substitution rate is itself time-modulated in an on-off fashion. In Huelsenbeck's model, evolutionary rates of sites, when in the on state, are modeled by a gamma distribution. Galtier [5] proposed a variant of this model, by merging the covarion-like random effects with the site-specific random-effects introduced by the RAS model: sites can take more than two rates ("on" and "off"), i.e. the off category plus, e.g., the four rates of a discretized gamma distribution. More recently, Wang et al. [31] propose a more general model in which evolutionary rates can switch among different rate classes when they are in a variable state.

One merit of Tuffley and Steel's version of the covarion model is that it aims at capturing the dynamic heterotachous scenario by using only two additional global stationary parameters: s_01_, the switching rate from the off to the on state, and s_10_, the rate from on to off.  Note that these two parameters are both assumed to be stationary over time. On the other hand, this model assumes that rate-shifts occur in a strictly site-independent fashion, whereas, in principle, it is possible to imagine more general scenarios, in which groups of sites undergo collective rate shifts at very specific time-points, due to a sudden change of the selection pressure (this type of situation is precisely supposed to create the misplacement of microsporidia [28,27]).

Recently, Kolaczkowski and Thornton [24] proposed a 'mixture of branch lengths' (MBL) model that could handle this kind of collective rate shifting. In this finite mixture model, which was later mathematically corrected [32], each observation is assumed to arise from one of several components (the number of components being predefined), each specifying a distinct and independent set of branch lengths, onto the same topology. Loosely speaking, each site can "choose" among the available components that which best describes its pattern of changes along the tree. In practice, as there is no a priori knowledge of which site belongs to which component, the likelihood at each site is a weighted sum over all components [33,32]. The kind of heterotachy assumed in the MBL model [24] can appear artificial at first sight, but is theoretically able to capture collective rate shifts, rather than the purely site independent on-off processes of the covarion model. In principle, the MBL model could thus provide a useful device for detecting singular and collective rate shift events.

However, the potential gain of the MBL over the covarion model is statistically expensive, because of the serious increase of the number of parameters implied (the number of additional branch lengths, (N_c_-1)*(2s-3), and the weights of the components, N_c_-1): (N_c_-1)*(2s-2), where N_c _is the number of components in the mixture, and s is the number of taxa. The MBL model poses practical challenges as well. For instance, in the Bayesian Markov chain Monte Carlo framework, the complicated structure of a single tree with several valuations (several sets of branch lengths) makes it difficult to propose update mechanisms that would be efficient for mixing in tree space, or, in a reversible-jump perspective, for averaging over the number of components. As a result, jointly estimating the phylogeny and the number of components will be a computational challenge.

A common statistical practice when facing computational difficulties is to make simplifying assumptions (e.g., a known phylogenetic tree), and to contrast the merit of different model configurations based on their statistical fit. Note that model comparisons based on likelihood ratio tests are not directly applicable here, as the set of models of interest do not all form a nested hierarchy. (Even evaluating the number of components would be difficult, because of the irregular parameter space in the mixture model [34,35], the logarithm of the likelihood follows a complicated mixture of chi-square distributions [36]). An alternative is to use likelihood penalty methods, such as the Bayesian Information Criterion (BIC; [37]), or Akaike Information Criterion (AIC; [38]). When the number of observations (here aligned sites) is sufficiently large, BIC is asymptotically equivalent to the Bayes factor, and AIC to the expected relative Kullback-Leibler information [38] Although easy to compute, these two measures rely on many assumptions to estimate the penalty for the increased number of parameters. Moreover, as for AIC, it further assumes that the models being tested are 'not too far' from the true model [38]. In addition, AIC seems to overestimate the number of parameters when there are many parameters compared to the sample size [39,40]. Contrary to AIC, BIC has a tendency to under-estimation, given sparse data and results [41]. Furthermore, in the context of mixture models, the regular assumptions for the AIC and BIC are no longer valid [42,43]. In any case, Djuric [44] argued that the penalty for over-parameterization should strongly depend on the model structure, i.e., the types of unknown model parameters. Although BIC works reasonably well at the practical level [45], Djuric [44] suggested a careful examination before applying AIC/BIC.

Another evaluation of model fitness is the cross-validation (CV) method [46]: it measures the predictive power of a model fitted to a first, randomly drawn, part of the dataset, when applied to the remaining (set aside) part of the data. Here, the portion of data set aside plays the role of 'future' observations. Accordingly, the best model is naturally the one that best predicts these future data. Compared to AIC and BIC, CV is computationally much more demanding, but also more reliable in principle: (1) this is an operational test, in which one measures the predictive power on data that have not been seen during the learning step, which guarantees the 'honesty' of the measure. In particular, it implies that there is no need to account for a dimensional penalty. (2) the expectation of cross-validated likelihood is an unbiased estimate of the Kullback-Leibler (KL) distance between the "true" distribution of column patterns, and the distribution implied by the model [47], and (3) in fairly general settings (not including the leave-one out testing scheme), cross validation is asymptotically consistent, i.e. is able to choose the true model among identifiable alternatives [48]. In addition to these theoretical guarantees, there is no specific requirement on the compared models (e.g. nested).

In this work, we explore the use of AIC, BIC and CV for the comparison of covarion and MBL models. We first validate and examine properties of the MBL model using simulations. Second, we contrast the conclusions of AIC, BIC and CV to the problem of determining the number of components of the MBL model, and to general comparisons with the covarion model. Third, we extend our model comparisons to three real data sets from nuclear, plastid and mitochondrial compartments, and show that the covarion model is always favored over the optimal MBL model.

## Results

### Simulated data

**Figure 1:**
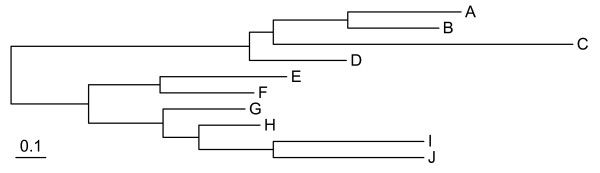
**Topology used for computer simulations**. The tree under the newick format is: ((((A:0.375, B:0.3):0.25, C:1):0.08, D:0.32):0.8,((E:0.42, F:0.31):0.24,(G:0.27,(H:0.2,(I:0.5, J:0.5):0.25):0.12):0.25):0.26). Scale bar indicates the expected number of changes per site.

We first implemented the mixture branch length model in the phylobayes package [49]. Simulations allowed us to explore the performance of the MBL model when the true number of components as well as other parameters are known. Various levels of heterotachy can be easily obtained by tuning a single parameter, *τ*, without affecting the average branch length (see Methods for details) of the tree topology displayed on Figure 1. In addition, the degree of rate variation across sites was modulated by using several values of *α*, the shape parameter of the gamma distribution. A total of 16 data sets of 5,000 sites each were synthesized under the two-component MBL model and analyzed using the MBL model with number of components varying from one to four.

When the simulated data are analyzed with the exact number of components (two), the inferred values of the parameters are generally close to their true values (Table 1). For instance, the value of *α *is always inferred with an error smaller than 5%. The branch lengths and the weights are also well recovered, although only when the level of heterotachy is pronounced (*τ*>= 0.4, Table 1). Interestingly, when weakly heterotachous datasets (*τ *= 0.2) are analyzed under the two-component model, the weight for one of the two components shrinks to almost zero, and the corresponding branch lengths become meaningless, taking on extremely large or small values.

**Table 1:** Inferred values of *α*, the parameter of the discrete gamma distribution of the rates across sites, inferred weight of one of the two components (w) and Pearson correlation (r) of the inferred tree branch lengths with the true ones of their respective component, for sequences simulated with various values for *τ *and *α*.

*α*/*w*/*r*	*τ *= 0.2	*τ *= 0.4	*τ *= 0.6	*τ *= 0.8
*α *= 0.5	0.51/0.028/n.a.	0.52/0.42/0.976	0.49/0.46/0.993	0.52/0.50/0.998
*α *= 1.0	1.06/0.033/n.a.	1.04/0.43/0.993	1.00/0.47/0.993	1.02/0.49/0.998
*α *= 1.5	1.51/0.07/n.a.	1.56/0.50/0.993	1.56/0.48/0.997	1.46/0.49/0.998
*α *= 2.0	2.01/0.005/n.a.	2.04/0.41/0.979	1.89/0.49/0.999	1.99/0.50/0.998

Note that the correlation between the true branch lengths of the two components are 0.86, 0.52, 0.19 and -0.16 with *τ *= 0.2, 0.4, 0.6 and 0.8, respectively. Two components were used for the inference. When *τ *= 0.2, the partition identity cannot be recovered, so the branch lengths cannot be compared with the true ones.

Inferring the number of components followed a similar, but more complex, pattern (Table 2). When the dataset contains a strong heterotachous signal (*τ *= 0.8), AIC, BIC and CV recover the expected number of components (two). In contrast, as the level of heterotachy gets weaker (*τ *= 0.2), all criteria almost always choose the one-component model. The amount of heterotachous signal is simply insufficient in these 5,000 positions. Interestingly, under these conditions, when the MBL model with two components is used, the weight of one of them tends to be extremely small (Table 1), which is consistent with the higher fit of the one-component model. For intermediate level of heterotachy (*τ *= 0.4 and 0.6), AIC supports 2 and 3 components and BIC 1 or 2, suggesting that AIC might tend to overestimate, and BIC might underestimate, the number of components, (Table 2). In contrast, in both cases, CV recovers the correct value.

**Table 2:** Optimal numbers of components determined by AIC, BIC or cross-validation (CV) on the simulated data with different levels of heterotachy (*τ*) and with different rate across sites heterogeneity (*α*).

AIC/BIC/CV	*τ *= 0.2	*τ *= 0.4	*τ *= 0.6	*τ *= 0.8
*α *= 0.5	1/1/1	2/1/2	2/2/2	2/2/2
*α *= 1.0	1/1/1	2/1/2	3/2/2	2/2/2
*α *= 1.5	2/1/1	2/2/2	2/2/2	2/2/2
*α *= 2.0	1/1/1	2/2/2	3/2/2	2/2/2

We next extended the comparisons by including the covarion model (Table 3). As expected because sequences were simulated using an MBL model, the covarion model is never favored. However, under a low level of heterotachy (*τ *= 0.2), the covarion model performs slightly better than the two-component model, in spite of the fact that the dataset is indeed a mixture of two components. This could be due to the fact that the covarion model requires less parameters than the 2-components MBL model.

**Table 3:** Cross-validation for the simulated datasets (*α *= 0.5)

	One component (homotachy)	Two-component	Three-component	Four-component	Covarion
*α *= 0.5					

*τ *= 0.2	0	10.5 ± 5.5	18.6 ± 7.9	20.6 ± 10.9	0.8 ± 2.4
*τ *= 0.4	2.0 ± 8.7	0	4.7 ± 9.4	14.7 ± 8.7	2.0 ± 8.6
*τ *= 0.6	84.5 ± 12.4	0	10.0 ± 7.2	21.9 ± 10.1	85.2 ± 12.9
*τ *= 0.8	359.5 ± 30.0	0	8.1 ± 6.5	15.9 ± 9.3	359.6 ± 29.4

*α *= 1					

*τ *= 0.2	0	9.6 ± 4.3	18.5 ± 9.1	23.8 ± 9.0	0.6 ± 1.9
*τ *= 0.4	13.0 ± 5.9	0	10.6 ± 4.4	17.3 ± 8.1	14.6 ± 5.3
*τ *= 0.6	101.4 ± 8.6	0	11.0 ± 6.0	18.1 ± 9.2	101.7 ± 8.4
*τ *= 0.8	472.0 ± 13.9	0	10.2 ± 5.5	13.6 ± 5.6	453.4 ± 14.0

*α *= 1.5					

*τ *= 0.2	0	11.7 ± 6.3	7.4 ± 4.4	18.4 ± 12.1	0.7 ± 1.8
*τ *= 0.4	36.6 ± 5.9	0	12.1 ± 7.1	18.9 ± 9.2	34.9 ± 5.4
*τ *= 0.6	136.7 ± 12.8	0	7.7 ± 6.3	15.9 ± 9.6	135.3 ± 12.7
*τ *= 0.8	505.6 ± 23.8	0	10.8 ± 7.6	19.1 ± 8.8	490.9 ± 24.5

*α *= 2					

*τ *= 0.2	0	11.2 ± 5.3	17.7 ± 10.4	26.1 ± 9.9	1.7 ± 2.4
*τ *= 0.4	37.5 ± 17.5	0	9.3 ± 11.6	18.6 ± 15.7	39.2 ± 18.5
*τ *= 0.6	173.9 ± 12.6	0	10.6 ± 4.6	12.4 ± 5.3	169.5 ± 12.0
*τ *= 0.8	596.1 ± 22.2	0	8.0 ± 1.5	15.1 ± 6.9	588.0 ± 23.0

The mean (± SD) of the difference between the CV log likelihood of the current model and the model with the highest CV log likelihood is given. Five random runs were performed for this two-fold CV.

### Real data

When applied to three real datasets from nuclear, mitochondrial and plastid compartments, CV and BIC always supports the covarion model (Table 4), while AIC favors parameter-rich MBL model. In the selection of the optimal number of components of the MBL model, CV always favors the two-component model (Table 4). In contrast, BIC favors one component, except for mitochondrial alignment in which four or six components are virtually indistinguishable (44,416.88 versus 44,416.75), and AIC three or four components.

**Table 4:** Comparison of the covarion model and MBL models with different number of components for three real datasets

	-LnL	AIC	BIC	CV
	Animal dataset (5,000 sites and 20 species)			

one-component	86468.5	86506.5	86630.3	82.1 ± 7.9
two-component	86302.7	86378.7	86626.4	37.8 ± 13.5
three-component	86222.7	86336.7	86708.2	47.9 ± 10.7
four-component	86167.6	86319.6	86814.9	69.0 ± 17.2
five-component	86126.8	86316.8	86936.0	82.2 ± 21.2
Six-component	86087.1	**86315.1**	87058.1	NC
covarion	86300.7	86340.7	**86471.0**	**0**
	plastid dataset (3,754 sites and 22 species)			

one-component	78225.2	78267.2	78398.0	75.3 ± 8.8
two-component	78056.4	78140.4	78402.1	34.2 ± 24.5
three-component	77996.7	78122.7	78515.2	49.8 ± 15.6
four-component	77925.8	**78093.8**	78617.2	60.3 ± 21.0
five-component	77926.2	78136.2	78790.4	72.4 ± 22.0
six-component	77900.4	78152.4	78937.5	NC
covarion	78070.9	78114.9	**78252.0**	**0**
	mitochondrial mammal dataset (3,591 sites and 17 species)			

one-component	44285.9	44317.9	44416.9	45.9 ± 3.7
two-component	44154.8	44218.8	44416.8	16.6 ± 7.5
three-component	44127.6	44223.6	44520.5	34.2 ± 12.3
four-component	44081.2	**44209.2**	44605.1	38.2 ± 15.4
five-component	44071.9	44231.9	44726.8	NC
six-component	44072.3	44264.3	44858.2	NC
covarion	44187.1	44222.1	**44330.4**	**0**

For CV, standard deviation can be easily computed and is thus indicated.

**Figure 2:**
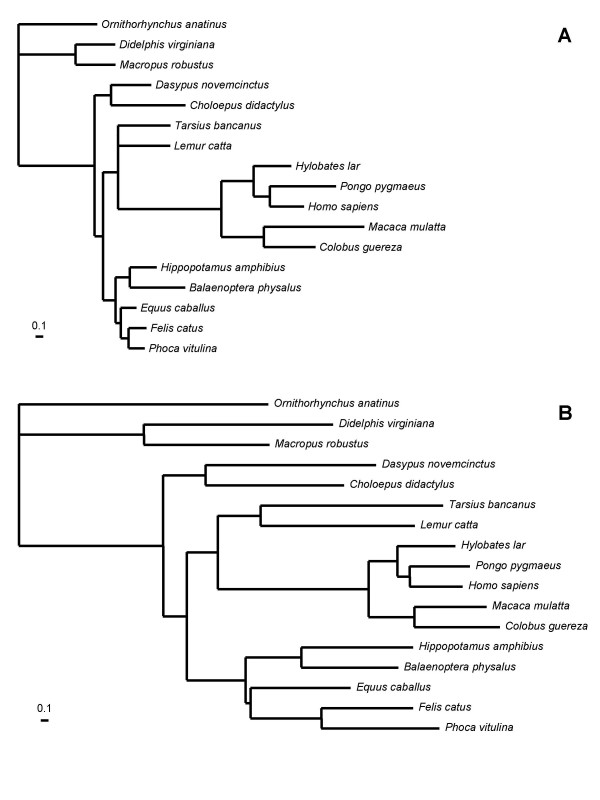
**Branch lengths for the two partitions in the case of the mitochondrial alignment of mammals (3591 sites, 17 species)**. The shape parameter of the Γ distribution was estimated to be 0.4. The weights are 0.40 for component I (B) and 0.60 for component II (A).

**Figure 3:**
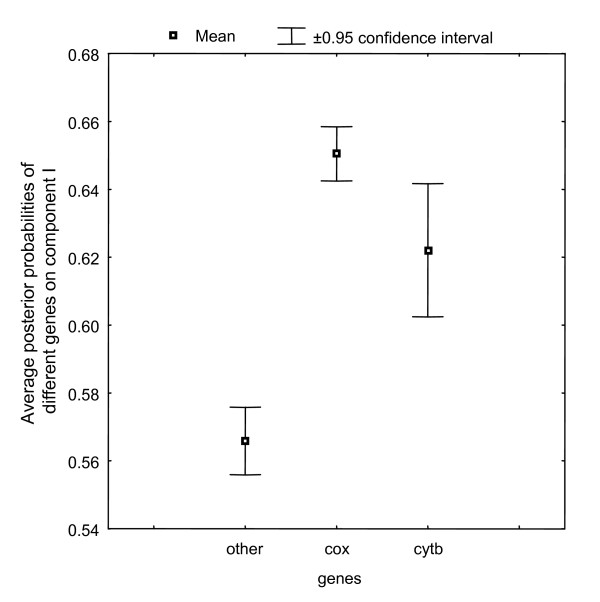
**Whiskers plot for the average posterior probabilities of component I for the two-component MBL model on the mitochondrial mammal dataset**. A Kruskal-Wallis non-parametric test shows the means of posterior probabilities for genes are significantly different (p < 0.0001)

We also studied the branch lengths of the two partitions detected by the MBL model (mitochondrial, Fig. 2; nuclear, see Additional File 1; plastid, see Additional File 2). Interestingly, in the case of mitochondrial alignment, the branch lengths of the two partitions mainly differ for catarrhinian primates, i.e. they evolved much faster in component I. To know whether particular genes are involved in this heterotachous behavior, we computed the posterior probability of each site belonging to either component (see Method, formula 9), and then averaged these posterior probabilities over the sites, separately for each gene. The sites belonging to the cytochrome oxidase (cox1-3) and cytochrome b (cytb) genes show a significantly different posterior probability of belonging to component I than the sites from other genes (P < 0.0001, Fig. 3). A chi-square test was also performed, showing that the two partitioning of the sites, into the cox/cytb or the non-cox/cytb gene groups, and into the 2 components of the model, are not independent (P < 0.001, Table 5). Similarly, for plastid alignment, the two components are biologically relevant. The branch lengths of one component are relatively clock-like whereas for the other one all green plants except *Mesostigma *showed a highly accelerated rate. Interestingly, RNA polymerases show a significantly higher posterior probability of belonging to component II than the sites from ribosomal proteins (P < 0.0001, see Additional File 3) in agreement with recent studies [50,22].

**Table 5:** Contingency table for the mitochondrial alignment

	Cox+Cytb	Other genes
Component 1	142/278	583/447
Component 2	1237/1101	1629/1765

Observed/expected numbers of positions are indicated.

## Discussion

### Model comparisons: CV is more reliable than AIC and BIC

The maximum likelihood value is always improved when more parameters are added to the model. The widely used likelihood penalty information criteria, AIC and BIC, evaluate the fitness of models by heuristically adjusting the likelihood score. Based on asymptotic arguments [37,38], they compensate for the automatic increase of the likelihood merely due to the increase in number of parameters, using simple (and distinct) formulae for the dimensional penalty. By construction, AIC gives a milder dimensional penalty than BIC. In many practical cases, the difference may be overwhelmed by the difference in log-likelihood between the two models. However, in the present case, and on both real and simulated data sets, AIC and BIC do not always reach the same conclusions (Tables 2 and 4).

Cross-validation methods are much more expensive in terms of CPU time than these information criteria. However, they are conceptually more trustworthy, since they consist in a true blind test, i.e. instead of relying on a heuristic dimensional penalty, they measure the predictive power of the model on data that have not been seen during the parameter optimization step. In addition, they are valid even far from the asymptotic regime, i.e. when the number of sites is small. From comparisons among AIC, BIC and CV, we observe that BIC and CV generally agree, while AIC overestimates the fit of parameter-rich models. These observations are consistent with the reports that AIC seems to have an inherent bias in favor of overly parameterized models [51-53,41,39,40],.

### Properties of the mixture branch length (MBL) model

The MBL model is able to detect heterotachous signals and recover the true number of components, sets of branch lengths, weights for the components, as well as the alpha parameter for the RAS gamma distribution, when datasets are simulated with a strong level of heterotachy (Tables 1 and 2). In contrast, when the level of heterotachy is weak (e.g. *τ *= 0.2) and the alignment size is in the order of magnitude of the currently used ones (5,000 amino acids), the homotachous (one component) model is preferred. This is consistent with the observations that the performance of the homotachous model is weakly affected under weakly heterotachous datasets (*τ *= 0.2), and that it starts to get devastating only when the level of heterotachy gets higher (*τ *= 0.4) [54,32,56,24]. It seems therefore that, at least on these simulated cases, when heterotachy does not impair phylogenetic inference, the classical non-mixture model is indeed found to be the optimal by standard model selection methods.

Estimating the adequate number of components can be viewed as a limitation of MBL models. On the one hand, we have shown that only the computationally demanding CV is able to provide an accurate estimate of the optimal number. On the other hand, it appears that, when the number of components is too high, the weights of these useless components are small (below 0.05, except for plastid -0.08- and nuclear -0.20- alignments). In other words, the over-parameterized model naturally reduces, but does not abolish, the effect of useless parameters, but is logically penalized in model comparison.

Interestingly, in the case of mitochondrial and plastid alignments, heterotachy detected by the MBL model is meaningful (Figs. 2 and S2). For instance, the most important heterotachous signal detected by the MBL model on the mitochondrial data set consists in a collective rate-shift, preferentially concerning the positions of cox and cytb gene. This acceleration of the multisubunit respiratory complex cytochrome c oxidase in primates is well documented and co-evolution implies genes encoded in the nucleus and in the mitochondrion [57]. Thus, the MBL model seems to be indeed able to detect collective behavior, corresponding to real biological events.

### How to model heterotachy?

However, and in spite of the considerable interest received by the MBL model recently [24,22,55,56,54,58,32], both BIC and cross-validation indicate that the covarion model performs significantly better than the MBL model on all real data sets we have analyzed so far. This considerably reduces the relevance of Kolaczkowski and Thornton (2004) observations, concerning the failure of current models and methods, including covarion, to correctly infer phylogenetic trees under heterotachous conditions, as it further confirms how artificial the simulation conditions were.

**Figure 4:**
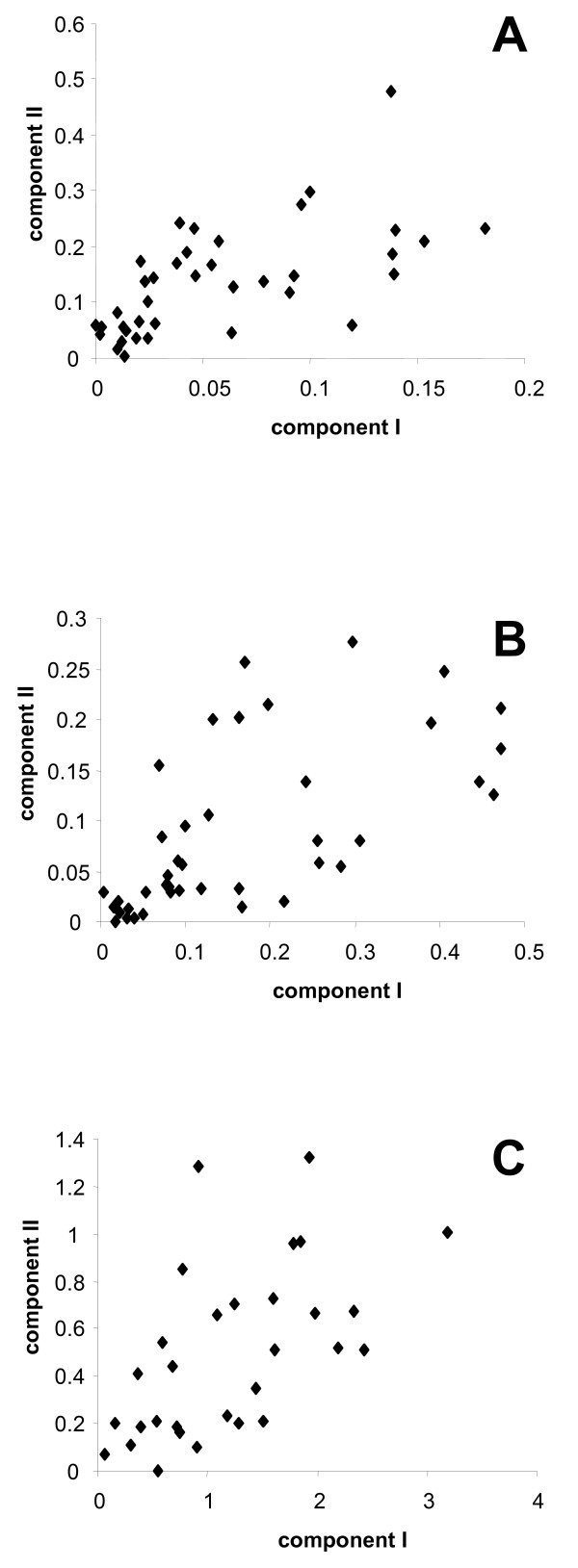
**Comparison of branch lengths from the two partitions for the nuclear (A), plastid (B) and mitochondrial (C) alignments**. R = 0.63, 0.63 and 0.57 respectively.

An obvious explanation for MBL's failure is that it is too parameter-rich ((N_c_-1) *(2s-2), s is the number of species and N_c _the number of components). Indeed, a completely new set of branch lengths has to be inferred for each component, which may be too expensive, as heterotachy may manifest itself only on a subset of the branches. Accordingly, branch lengths of the two components are relatively well correlated (R between 0.57 and 0.63, Fig. 4), illustrating a parametric redundancy. The difference in the behavior of the covarion model and the MBL model on the real datasets and the simulation datasets implies that the real dataset might not have such global rate shifts (i.e. all the corresponding branch lengths in different categories would be drastically different) as designed in the simulation datasets.

When multiple genes are analyzed, a separate model [59] is aimed at capturing heterotachous signal among genes. The only difference with the MBL model is that the number of components and their structures are defined a priori. The separate model may therefore probably suffer from the same weaknesses as the MBL model, an inherent over-parameterization due to the fact that branch lengths are well correlated among genes, with few exceptions [60]. On the other hand, it may lead to more accurate phylogenetic inference, in case where the covarion model failed [50]. This indicates that both the separate model and MBL-like approaches still deserve further studies.

Mixture models generally imply numerous additional parameters. Improved fitness is obtained only if most of these additional parameters are natural, i.e. have a great explanatory power. This is for example the case for the CAT model [7] in which components reflect the amino acid spectrum allowed by structural and functional constraints. Unfortunately, the combinatorial effect is too important for MBL modeling to be efficient for instance, assuming only 2 independent collective rate shifts on two distinct branches, involving two intersecting groups of sites, will create 4 distinct site patterns, describing all possible ways a given site may have 'responded' to the first and/or to the second rate shift. In this situation, the MBL model will need 4 components to explain every site correctly. More generally, with S independent collective rate shifts, 2^S ^components will be needed to describe all possible combinations that will all be likely to occur across the alignment. This combinatorial argument may explain the failure of the MBL model in practice, in spite of its ability to detect collective behaviors.

## Conclusion

The covarion model, in spite of its better fit, is a purely site-independent model. As such, it may not be optimally efficient at capturing collective rate shifts, such as those that we can detect using MBL, and may instead be meant for the background of "stationary" heterotachy present at every site. This suggests that an explicit model accounting for collective events, in the spirit of MBL, albeit more parsimonious in terms of parameterization, would be an interesting direction to take. A natural approach to do this would be a divergence point model [61-63], where, due to the functional and/or structural shift, some sites evolve differently from other sites in the different areas of the phylogeny defined by the divergence points.

In another direction, the covarion model, in the version that we test here [6], can also be improved. Wang et al. [31] introduced a more general model, in which rate can not only switch from on to off but also from a given rate to another and demonstrated a slight, but generally significant, improvement. Yet, this model remains homogeneous over positions, a constraint that could be released by considering a mixture model in which the parameters of the covarion process are component specific.

## Methods

### The mixture branch length (MBL) model

The mixture model assumes several components with different sets of branch lengths. When sites are assumed to be independent, the likelihood for the data *D *in the mixture model is the product of *N *site-specific likelihoods, and each site's likelihood is the sum of likelihoods over all *Nc* components, weighted by the components' probabilities *w *$(\sum_{k=\text{1}}^{Nc}w_{k}=\text{1}):$

$$\begin{array}{l} P(D|l,w,\tau ,\theta )=\prod_{i=\text{1}}^{N}\sum_{k=\text{1}}^{Nc}w_{k}P(C_{i}|l_{k},\tau ,\theta ) \end{array}$$

Where *l *is *Nc *sets of (2s-3) branch lengths (s is the number of species); *τ *is the topology; *θ *is the rest of parameters (such as rate matrix, stationary probability); and *C*_*i *_is the alignment column at site *i*. The MBL model is implemented based on a homemade software, which uses a Bayesian Markov chain Monte Carlo (MCMC) sampler [7]. Maximum likelihood was calculated via simulated annealing.

### The covarion model

 The covarion model corresponds to a doubly stochastic process The   process of rate switching is described as:

$$S=\left[\begin{array}{cc} -s_{01} & s_{01}\\ s_{10} & -s_{10} \end{array}\right]$$

where s_01 _is the rate of switching from off to on; s_10 _is the rate of switching from on to off. Thus, two parameters are necessary for this process, the rates of switching between the two states, off and on. When a site is in the on state, it   undergoes substitutions among the 20 amino-acids according to a first   order Markov process, described by a rate matrix Q. Here, for both the   covarion and MBL models, this substitution process was described by a   JTT+Γ model with four discrete categories..

The rate matrix can be

$$R=\left[\begin{array}{ll} -s_{01}I & s_{01}I\\ s_{10}I & Q-s_{10}I \end{array}\right]$$

where I is the identity matrix (r × r, r is the number of states, for a protein data set, r = 20). For more details on the implementation, see refs. [30] and [6].  Therefore, R is 40 × 40 rate matrix for the covarion in the Markov process. For both the MBL and covarion models, the substitution process was described by a JTT+Γ model with four discrete categories.

### Maximum likelihood estimation using simulated annealing

We use simulated annealing, within our MCMC sampler, to obtain the maximum likelihood estimation. Simulated annealing is a straightforward generalization of the MCMC algorithm, especially for high-dimensional models such as MBL [64]. In a normal MCMC run, at each cycle, a new parameter value (*x*'), slightly different from the current one (*x*), is proposed according to a stochastic kernel q(*x*, d*x*'), and accepted according to the Metropolis-Hastings rule, i.e. with probability

$$\alpha (x,x')=\text{min}\left\{\text{1, }\left[\frac{L(x')}{L(x)}\right]\left[\frac{q(x\text{',}dx)}{q(x,dx')}\right]\right\}$$

where *L(x) *is the likelihood for the current state; *L(x') *is the likelihood for the proposed state; *q(x', dx) *is the probability of proposing from *x*' to *dx *state; *q(x, dx') *is the probability of proposing from *x *to *dx' *state. The only additional feature to be implemented for simulated annealing is to replace this Metropolis Hastings version by its thermal version:

$$\alpha (x,x')=\text{min}\left\{\text{1, }{\left[\frac{L(x')}{L(x)}\right]}^{\beta }\left[\frac{q(x\text{',}dx)}{q(x,dx')}\right]\right\}$$

Here, *β *is analogous to an inverse temperature. If *β*<1, the Markov chain is heated up (the equilibrium distribution is flatter than the posterior distribution), and if *β*>1, it is cooled down (the equilibrium distribution is more peaked around its mode). At the reference temperature (*β *= 1), it reduces to the posterior distribution.

Based on this modification of the Metropolis principle, one can mimic the process of a thermodynamic annealing to obtain the maxima: we start at a high temperature (*β *= 1), whereby the posterior distributions are extensively visited; then, as the temperature decreases (as *β *increases), the distribution explored by the MCMC gets progressively more peaked around the mode, until, at a sufficiently low temperature, the Markov chain "freezes" at the ML estimate. Our cooling schedule consists in starting with *β *= 1, and increasing its value geometrically (i.e. *β *= 1.01* *β*), until *β *= 50000. To check whether the chain gets stuck in local maxima, several independent runs with random starting points are performed, and compared with each other. All the independent runs were found to converge at the same maximal point.

### Model evaluations

The BIC [37] is defined as:

$$\text{BIC}=-\text{ln}p\left(D|\hat{\theta }\right)+\frac{K\text{ln}N}{\text{2}}$$

where $\hat{\theta }$ is now the overall set of parameters maximizing the log-likelihood ln*p*(*D*|$\hat{\theta }$), *K *is the number of parameters that have been adjusted in $\hat{\theta }$, and *N *is the number of sites. The penalty depends both on the number of parameters and on the number of sites; the smaller the BIC, the better the fitness of the model. Another criterion similar to the BIC, but less strict, is the Akaike Information Criteria (AIC; [38]), for which the penalty only depends on the number of parameters:

$$\text{AIC}=-\text{ln}p\left(D|\hat{\theta }\right)+K$$

A second order correction for the AIC [65] has a negligible impact in the present context, and so is not reported here.

We also compared models by the cross-validation (CV). Briefly, for a given model, we first optimize parameters on a portion of the dataset, i.e. the *learning set *(*D*_*L*_), then use these parameters ($\hat{\theta }$_*L*_) to compute the likelihood of the *testing set (D*_*T*_). Thus, the CV score is obtained by sampling the *learning set *and the *testing set *several times, and taking the expectation of the likelihood over these replicates (parameters being inferred from the training tests):

$$\text{CV}=E\left[-\text{ln}p\left(D_{T}|{\hat{\theta }}_{L}\right)\right]$$

By averaging over replicates, one gets rid of sampling errors in the partitioning of the dataset into a learning set and a test set. In particular, one smoothes out possible (albeit unlikely) uneven repartitions in which sites corresponding to distinct components of the mixture would be partially segregated.

The *learning set *(*D*_*L*_) and the *testing set (D*_*T*_) can be created in various ways. One method is the so-called *v-fold cross-validation*. The original data set is partitioned into *v *disjoint subsets of equal size; then each partition is successively used as the *testing set (D*_*T*_), the union of all other *v*-1 partitions being used as the *learning set *(*D*_*L*_). The overall procedure is repeated until a total of *v *tests have been performed. In this work, we used the most currently used 2-fold cross-validation schemes. The random sampling of half data set was performed ten times, which yielded a precision of CV score sufficient to discriminate among the models under study. This small value is therefore a good compromise between computational time and accuracy.

### Identifying the optimal component for each site

Since we do not know exactly which component a given site belongs to, the likelihood for one site is the weighted sum of likelihoods conditional on each possible allocation of the site to the available components. We can, however, calculate the posterior probability of a site (i) belonging to a given component (k):

$$P(l_{k}|C_{i})=\frac{w_{k}P(C_{i}|l_{k})}{\sum_{k=\text{1}}^{Nb}w_{k}P(C_{i}|l_{k})}$$

These posterior probabilities were then averaged over the sites, for each gene of the alignment. Alternatively, each site was affiliated to the component of higher posterior probability, and a chi-square test of the independence between the affiliations to the component, and the affiliation to each of the genes, was performed.

### Simulations

All the simulations were done with the JTT replacement matrix, rate across site heterogeneity being modeled by a Γ distribution (four discrete categories). Heterotachous data were simulated by concatenating two alignments generated under the same tree topology, but with different branch lengths [24,54]. Briefly, a reference tree, with branch lengths specified, is chosen (Fig. 1). Next, each branch length of the two partitions is adjusted by multiplying the length of the reference tree either with (1 + *τ*), or with (1 - *τ*), where *τ *∈ [0,1] is a parameter tuning the extent of heterotachy. The choice between the two opposite multipliers ((1 + *τ*) and (1 - *τ*)) is made at random, independently for each branch while under two constraints: a) the corresponding branch in the two partitions should be adjusted with opposite multipliers; b) in one partition, sister branches should be adjusted with opposite multipliers also; i.e., if one branch length in one partition is increased by a factor (1 + *τ*), then the same branch in the other partition is decreased by a factor (1 - *τ*) and also the sibling branch length in the same partition is decreased by a factor (1 - *τ*). In this way, the average length over the alignment remains equal to the reference length [54] and the branch length heterogeneity strictly followed the strategy by Kolaczkowski and Thornton [24], i.e., the branch lengths in each component tend to behavior in a Felsenstein zone. Totally, 16 simulated datasets are generated with different discrete *α *(0.5,1,1.5,2) and different *τ*(0.2,0.4,0.6,0.8).

### Real Datasets

Three protein datasets were used to examine the fitness of the covarion model, the mixture branch length models, and the homotachous model (one-component model):

• Nuclear alignment: a subsample was obtained from the dataset of 133 nuclear genes and 57 animal species [66]. The twenty most complete species were selected. For computing time reason, only the first 5000 sites were used.

• Plastid alignment: the dataset was created by concatenating plastid ribosomal proteins (rpl14, rpl20, rpl2, rpl33, rps12, rps16, rpl16, rpl22, rpl32, rpl37, rps19, rps3, rps7, rps11, rps14, rps18, rps2, rps4 and rps8) and RNA polymerase proteins (rpolA, rpolBp, and rpolB) from green plants, glaucophytes, red algae, cryptophytes, stramenopiles and haptophytes. The ambiguously aligned regions were removed using Gblocks [67]. The final alignment contains 22 species and 3754 sites.

• Mitochondrial alignment: we used a concatenation of 12 mitochondrial genes (atp6, atp8, cox1, cox2, cox3, cytochrome b, nad1, nad2, nad3, nad4, nad4L and nad5) totally 3591 sites from 17 mammals.

The computing times for a CV replicate (on Pentium P4, 3.2 GHz) are approximately 80 and 190 (MBL 2 components and covarion), 40 and 110, and 35 and 80 hours for nuclear, plastid and mitochondrial datasets, respectively.

## Authors' contributions

YZ implemented the covarion and MBL models into phylobayes, made all the computations, and wrote the first draft of the manuscript. NL and NR helped in the programming and MCMC settings. HP and NL conceived and supervised the study. All authors contributed to the analysis of the results and to the writing of the paper. They read and approved the final manuscript.

## Supplementary Material

Additional file 1 MBL model in the case of the nuclear alignment of opisthokonts.The branch lengths for the two partitions are provided. Click here for file

Additional file 2 MBL model in the case of the plastid alignment of plants. The branch lengths for the two partitions are provided. Click here for file

Additional file 3 MBL model and gene function in the case of the plastid alignment of plants. Average posterior probabilities of component I for the two-component MBL model are provided. Click here for file
