# Supplementary material for: Evaluation of the models handling heterotachy in phylogenetic inference
Source: BMC Evol Biol. 2007 Nov 1;7:206. doi: 10.1186/1471-2148-7-206 (PMC2248194; doi:10.1186/1471-2148-7-206)
Supplement: Additional file 1 — MBL model in the case of the nuclear alignment of opisthokonts.The branch lengths for the two partitions are provided. [file 1471-2148-7-206-S1.ppt]

## Slide 1
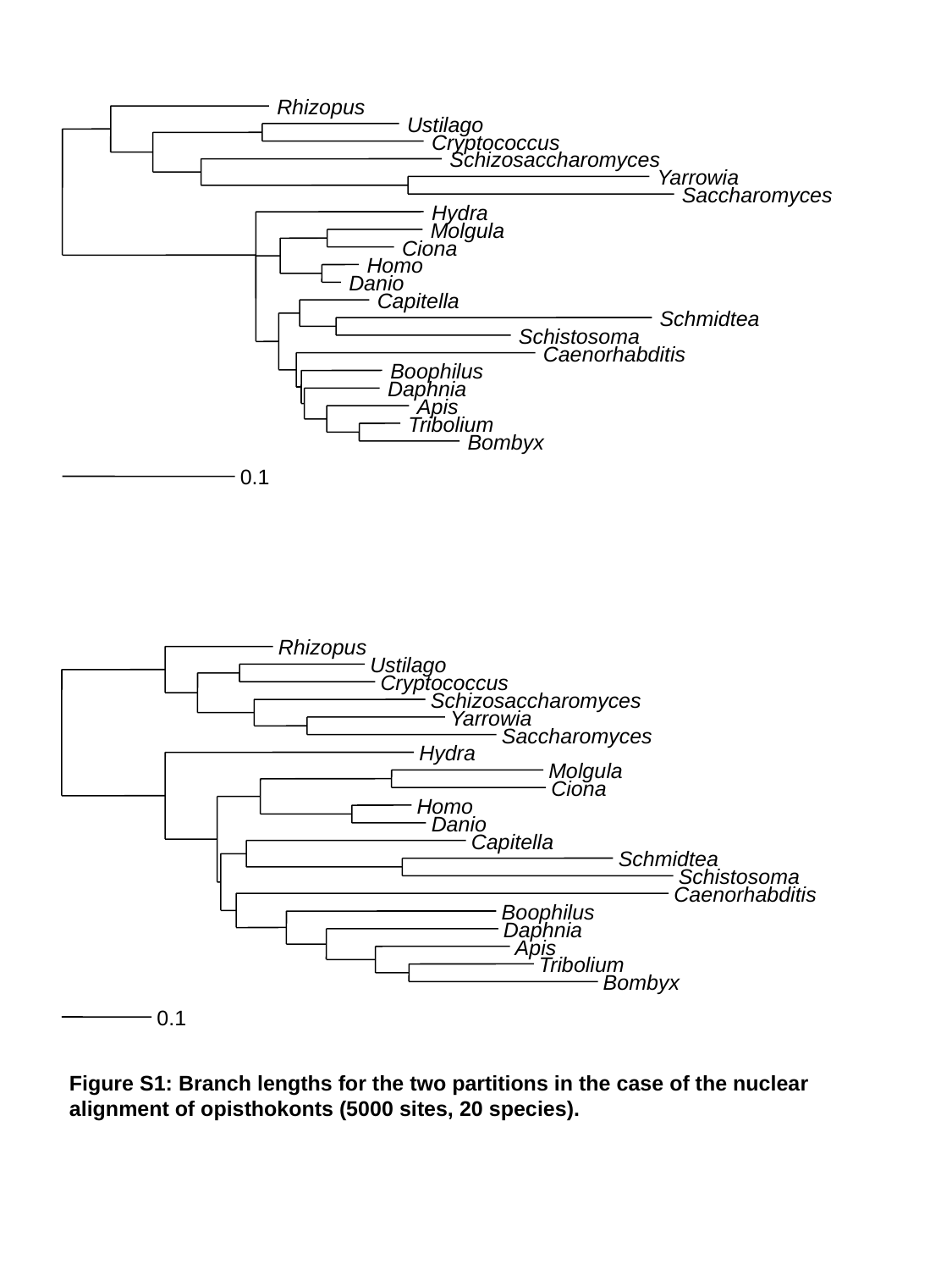

Rhizopus
Ustilago
Cryptococcus
Schizosaccharomyces
Yarrowia
Saccharomyces
Hydra
Molgula
Ciona
Homo
Danio
Capitella
Schmidtea
Schistosoma
Caenorhabditis
Boophilus
Daphnia
Apis
Tribolium
Bombyx
0.1
Rhizopus
Ustilago
Cryptococcus
Schizosaccharomyces
Yarrowia
Saccharomyces
Hydra
Molgula
Ciona
Homo
Danio
Capitella
Schmidtea
Schistosoma
Caenorhabditis
Boophilus
Daphnia
Apis
Tribolium
Bombyx
0.1
Figure S1: Branch lengths for the two partitions in the case of the nuclear alignment of opisthokonts (5000 sites, 20 species).
