# Supplementary material for: Evaluation of the models handling heterotachy in phylogenetic inference
Source: BMC Evol Biol. 2007 Nov 1;7:206. doi: 10.1186/1471-2148-7-206 (PMC2248194; doi:10.1186/1471-2148-7-206)
Supplement: Additional file 2 — MBL model in the case of the plastid alignment of plants. The branch lengths for the two partitions are provided. [file 1471-2148-7-206-S2.ppt]

## Slide 1
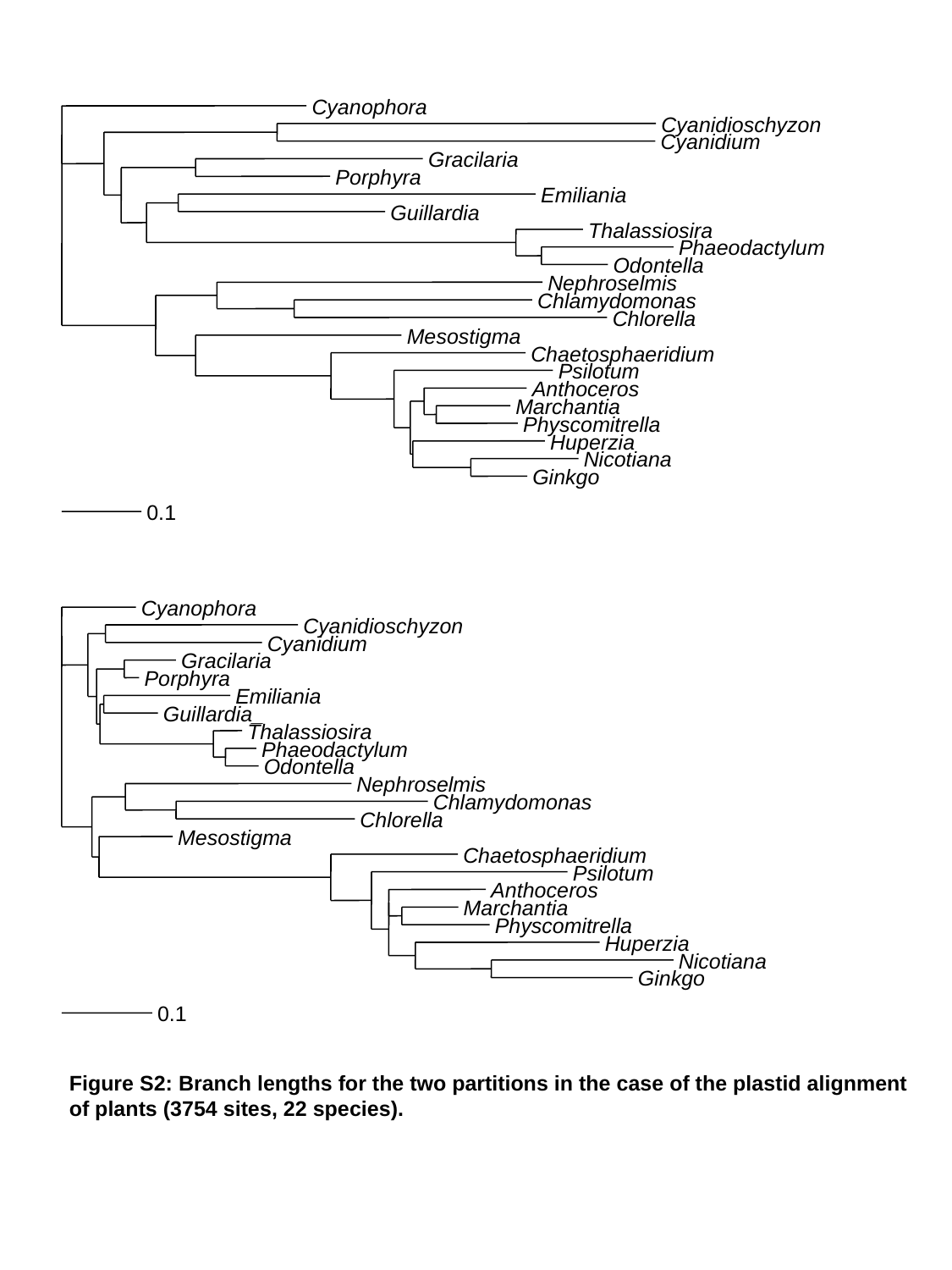

Cyanophora
Cyanidioschyzon
Cyanidium
Gracilaria
Porphyra
Emiliania
Guillardia
Thalassiosira
Phaeodactylum
Odontella
Nephroselmis
Chlamydomonas
Chlorella
Mesostigma
Chaetosphaeridium
Psilotum
Anthoceros
Marchantia
Physcomitrella
Huperzia
Nicotiana
Ginkgo
0.1
Cyanophora
Cyanidioschyzon
Cyanidium
Gracilaria
Porphyra
Emiliania
Guillardia_
Thalassiosira
Phaeodactylum
Odontella
Nephroselmis
Chlamydomonas
Chlorella
Mesostigma
Chaetosphaeridium
Psilotum
Anthoceros
Marchantia
Physcomitrella
Huperzia
Nicotiana
Ginkgo
0.1
Figure S2: Branch lengths for the two partitions in the case of the plastid alignment of plants (3754 sites, 22 species).
