# Supplementary material for: Evaluation of the models handling heterotachy in phylogenetic inference
Source: BMC Evol Biol. 2007 Nov 1;7:206. doi: 10.1186/1471-2148-7-206 (PMC2248194; doi:10.1186/1471-2148-7-206)
Supplement: Additional file 3 — MBL model and gene function in the case of the plastid alignment of plants. Average posterior probabilities of component I for the two-component MBL model are provided. [file 1471-2148-7-206-S3.doc]

**Table S1: Average posterior probabilities of component I for the two-component MBL model on the plastid dataset.**

|  | Mean (SD) |
| --- | --- |
| LSU ribosomal proteins (867 positions) | 0.506 (0.27) |
| RNA polymerases  (1547 positions) | 0.589 (0.25) |
| SSU ribosomal proteins (1340 positions) | 0.513 (0.27) |
